# Personal

**Published:** 1896-05

**Authors:** 


### Personal.

---

Dr. Arthur G. Bennett, of Buffalo, whose practice is limited to diseases of the eye and ear, has removed from 213 Franklin street to 191 Delaware avenue. Hours, 9-1 and 4 P. M.

---

Dr. H. E. Hayd, of Buffalo, has removed from 78 Niagara street to 493 Delaware avenue.

---

Dr. F. G. Moehlau, of 1298 Jefferson street, Buffalo, will sail for Europe, May 9, 1896. He will spend four months at the universities of Erlangen and Berlin in study of special departments of medicine, to the practice of which he intends to devote himself in future.

---

Dr. Melancthon Storrs has been elected president of the Hartford medical society for the ensuing year. Dr. Storrs is one of the most eminent physicians in New England.

---

Dr. J. H. Carstens, of Detroit, read a valuable paper before the Alumni Association of Trinity University, Toronto, Ont., April 7, 1896. He also made an appropriate speech at the annual banquet, held in the evening at the Hotel Rossin. These international exchanges of courtesy are beneficial in their influence.

---

Dr. A. Walter Suter, of Herkimer, N. Y., in a brief note to the medical society of the State of New York, showed that recent experiments with bullets infected with the bacillus anthrax proved that the heat produced by the friction did not, as might have been expected, sterilise the projectile, and hence this opened up an additional source of wound infection.

---

DR. GEORGE H. ROHÉ has resigned as superintendent of the Maryland Hospital for the Insane, at Catonsville, usually called "Spring Grove," and will take charge of the new insane hospital to be built at Springfield, Md.

---

HON. MELVIL DEWEY, secretary of the board of regents, University of the State of New York, has been exonerated from all suspicion of irregularity or illegality in the conduct of his office. The investigating committee of the legislature made its report to the assembly April 24, 1896, in which it states that not a single charge was sustained. Those who know Mr. Dewey will not be surprised at this result, though they were amazed at the maliciousness in which the charges were conceived and pursued.

